# Epidemiology of Familial Amyloid Polyneuropathy in Bulgaria

**DOI:** 10.1186/1750-1172-10-S1-O2

**Published:** 2015-11-02

**Authors:** Stayko Sarafov, Mariana Gospodinova, Velina Guergueltcheva Velina, Andrei Kirov, Chamova Teodora, Tihomir Todorov, Alebena Todorova, Ivailo Tournev

**Affiliations:** 1 Sofia, Bulgaria

## Background

TTR FAP was diagnosed in 2008 for first time in Bulgaria. Molecular analysis is available since 2010.

## Methods

Four mutations have been found in the country so far: Glu89Gln, Ser77Phe, Val30Met, Ser52Pro. Glu89Gln is the most frequent mutation affecting 48 different families. Selective genetic screening program is performed in the affected families. A total of 261 individuals belonging to affected families were examined. All individuals who took part in the screening program signed an informed consent for a voluntarily participation in the program. TTR-FAP mutations were found in 130 individuals: Glu89Gln – 107; Val30Met – 15; Ser77Phe – 5; Ser52Pro – 2; compound heterozygous carrier of two mutations: Glu89Gln and Val30Met – 1.

## Results

Most of the FAP families with Glu89Gln mutation are concentrated in two districts of the South-West part of the country on the boundary with Macedonia. We accept this mutation as typical for the Balkan – Mediterranean region with possible founder effect connecting patients with Glu89Gln from neighbouring countries. Ser77Ple is presented in 5 families originating from only one village. In a historical view we do not exclude inserting of this mutation in our population century ago. Val30Met is relatively rare till now in Bulgaria. Most of the patients originate from small focus placed in the south-east part near the border with Greece.

## Conclusion

Every one of the mutations presents with specific clinical phenotype as: average age of onset, disease course / survival, male / female distribution, first symptoms – polyneuropathy, cardiac, gastro-intestinal or other involvement, sporadic or familial. The clinical phenotype and the place of origin are helpful for preliminary information for the expected mutation.

